# Who moved my scan? Early adopter experiences with pre- and post-market healthcare AI regulation challenges

**DOI:** 10.3389/fmed.2025.1651934

**Published:** 2025-08-26

**Authors:** Aviad Raz, Yael Inbar, Netta Avnoon, Liat Bela Lifshitz-Milwidsky, Barkan Hofman, Asaf Honig, Ziv Paz

**Affiliations:** ^1^Department of Sociology and Anthropology, Ben-Gurion University of the Negev, Be’ersheba, Israel; ^2^Technology Management and Information Systems, Coller School of Management, Tel Aviv University, Tel Aviv-Yafo, Israel; ^3^Western University, London, ON, Canada; ^4^Clalit Health Services, Tel Aviv-Yafo, Israel

**Keywords:** AI, explainability, healthcare, regulation, human-AI teaming, early adoption, XAI

## Abstract

Real-world clinical experience provides a much-needed opportunity for deep learning AI algorithms to evolve and improve. Yet, it also constitutes a regulatory challenge, since such potential for learning may essentially change the algorithm and introduce new biases. We focus on the gaps between “lifecycle” regulation and implementation from the perspective of the deployers, addressing three interconnected dimensions: (a) How precautionary regulation affects AI deployment in healthcare, (b) How healthcare providers view explainable AI (XAI), and (c) How AI deployment influences, and is influenced by, team routines in clinical settings. We conclude by suggesting ways in which the ends of healthcare AI regulation and deployment can successfully meet.

## 1 Introduction

The advent of potential artificial intelligence (AI) automation in healthcare paradoxically leads to a growing need for human supervision both before and after deployment (1). In contrast to conventional, static medical devices, the unique power of AI and specifically machine learning (ML)-based algorithms lies in their ability for dynamic, continuous learning, before and after deployment. Real-world experience evidently provides an important opportunity for evolving and improving for such deep learning algorithms. Yet, it also constitutes a regulatory challenge, since such potential for learning may essentially change the algorithm and introduce new biases (2). To avoid re-approval by the US Food and Drug Administration (FDA), the learning capability of many healthcare AI systems is shut down upon deployment, thus removing the risk along with the potency. The challenge is, therefore, how to match such adaptive systems with an equally adaptive regulation? There is little theorization, let alone research, addressing the gap between the design of AI “lifecycle” regulation and its practical application in clinical settings, including the role of healthcare providers as early AI deployers. This Perspective Article sets out to fill some of these literature voids.

According to the new EU AI Act (EU AIA), healthcare AI falls under the “high-risk” category and requires oversight by deployers. Yet, clarification is needed regarding how exactly should deployers oversee such “adaptive” or “self-learning” systems. Additionally, as deployers encompass both healthcare organizations and individual clinicians, clarifying this distinction is important as the challenges and motivations for each can differ. Recognizing this challenge, the US FDA is in the process of shifting from the traditional medical device regulatory framework and has introduced the AI/ML-based software as a medical device action plan in 2021, employing a “total product lifecycle” approach to include post-market phases–covering data quality, algorithm transparency, and robust change management (3). In 2024, the FDA further issued guidelines for an adaptive regulatory framework of AI/ML-enabled device software (4, 5). This framework potentially lets developers address AI’s continuous evolution without repeatedly seeking new FDA approvals. However, on-site post-deployment evaluations of AI in diverse clinical settings, requiring the implementation of robust, real-time monitoring mechanisms, remain inconsistent and underdeveloped (2, 6). This often means that healthcare providers-cum-deployers must step in to develop local protocols for quality assurance and recalibration.

To address these challenges of pre- and post-market regulation we focus here on the gaps from the perspective of the deployers. Looking at AI regulation in healthcare from the point of view of deployers, we focus on three interconnected dimensions: (a) how precautionary regulation affects AI deployment in healthcare, (b) how healthcare providers’ assumptions about explainable AI (XAI) relate to clinical trust, and (c) how AI deployment influences, and is influenced by, team routines in clinical settings. We conclude by suggesting ways in which the ends of healthcare AI regulation and deployment can successfully meet.

## 2 How precautionary regulation affects AI deployment in healthcare

Food and Drug Administration and the EU-AIA regulatory frameworks focus on pre-market approval and validation yet largely fail to provide a workable solution regarding the post-deployment need for continuous monitoring, re-training and re-validation of AI models (2). Scholars have warned that as AI models are exposed to new data in clinical settings, their performance may degrade or alter overtime, necessitating ongoing oversight (7). Recognizing this, the FDA asked developers to be accountable for the real-world performance of their AI systems and update the FDA on the changes in terms of performance and input (8). The significant financial and administrative requirements implied by such re-assessment almost always lead to avoiding system retraining, or re-validation, after deployment, compensated by selective use patterns. An illustrative case is ChestLink, an X-ray imaging AI, which in 2022 was the first fully autonomous healthcare AI to receive the EU-CE mark certificate, including post-deployment AI re-training. The catch is that ChestLink is only allowed to autonomously produce reports for healthy patients (9). It does not autonomously analyze or report on chest X-rays with abnormalities. Pooling real-world evidence from an adaptive Chestlink could have shown the trade-off between the risk reduced by suppressing its utility versus letting it learn from diagnosing all cases, under clinicians’ supervision.

The need for *in situ* customization of healthcare AI tools gives way to experimental strategies, including vendor-appointed “champions” selected from healthcare deployers as well as on-site feedback loops, with physicians taking the role of the “AI’s QA testers,” figuring out in which areas it is good enough and where it is over-sensitive and over-alerting due to “factory settings.” Nevertheless, such customization requires further systematic and standardized monitoring of bias metrics in model outputs and selective use by physicians.

Recent EU regulation also asks for AI transparency and explainability. Art. 13 of the EU AIA states that sufficient transparency shall be provided to those using the system under their authority (10). Art. 86 of the AI Act (Right to explanation) states that patients have the right to obtain clear and meaningful explanations of the role of the AI system in the decision-making procedure from the deployer (10). However, such provisions may be interpreted as a right to a partial explanation only of the role of AI, rather than its functioning. The lack of specific regulation concerning explainability, reflecting the lack of consensus on what it should consist of, may lead to inadvertent consequences, as explored in the next section.

## 3 How healthcare providers view explainable AI (XAI)

Defining explainability is a debated issue. Defined by one expert group as “the ability to explain both the technical processes of an AI system and the related human decisions” (11), others see explainability more broadly as a combination of both intelligibility and accountability (12). Transparency, closely related to explainability, is seen by some as a more passive characteristic of the system (13), while others claim that transparency (rather than explainability) relates to the initial training dataset and system architecture (14).

Is explainability a necessary condition for trust? Practically, the value of explainability requires a trade-off, e.g., with accuracy (15). At the relatively early stage of deployment that we are in, healthcare professionals arguably have significantly limited trust in AI tools, especially as they are charged with their QA. Explainable AI (XAI) was found to have the same influence on doctors’ decision-making as AI by itself (16). Nevertheless, explainability is arguably a liability issue. If the AI makes a mistake that causes harm to a patient, who is responsible? In addition, the opaque nature of AI/ML may hinder the conveyance of predictions to the patient, creating in the future a potential flood of medical malpractice lawsuits. Thus, clinicians’ preference for AI accuracy rather than explainability may very well change as liability models evolve, increasing the demand for XAI as a tool for creating a defensible record of clinical decision-making.

Expecting every clinical AI system to provide an explanation of its actions imposes a considerable limitation. Contemporary AI technologies markedly outperform older AI technology but are often not able to provide explanations understandable to humans. Regulators do not currently require human-comprehensible explanations for AI in other industries that have potential risks, such as autonomous vehicles. Health providers are often more concerned with the prediction power and efficiency of AI than with its explainability, especially due to time constrains and the need to be efficient with the exponential increase in the workload worldwide.

Post-deployment, a pragmatic shift is arguably emerging from explainability as an inherent characteristic of an algorithm, to explainability as an emergent and approximated property, increasingly tweaked around prediction power. In image detection algorithms, usually Convolutional Neural Networks, their first layers will contain references to shading and edge detection. The human doctor might never have to explicitly define an edge or a shadow, but their clustering may be referred to as “Grade X occlusion” of a blood vessel. The recent suggestion (17) to train medical AI to speak in “higher level” diagnostic concepts, such as “stage 1 cancer” rather than “X density of pixels in location Y,” arguably promoting more explainability and therefore more trust among deployers, reflects certain pre-assumptions regarding the perceived positive value of XAI and the dynamics of clinical teamwork. Is there really a “crisis of explainability” (other than by its legal demand), that leads to distrust by physicians of clinical AI?

Physicians may also be critical of letting AI make clinical recommendations, acknowledging that “an alert” might “make you think of [an] alternative diagnosis” but should not “sway” or “influence” the clinician. From a perspective of professional jurisdiction, it is to be expected that physicians insist on retaining the AI system as a “second pair of eyes” which should not disrupt the clinician’s authority (18).

Regulation of healthcare AI also forbids its full autonomy because of the potential risk assumingly involved in crossing the line between autonomous AI-driven diagnosis and augmented AI-supported human decision-making. Consequently, some medical imaging AI systems may highlight pixels on the scan (“first order” data) but not circle around these pixels (“second order” interpretation); or present predictions (“% of recurring cancer”) which doctors do not share with patients because of their opaqueness.

## 4 How AI deployment influences, and is influenced by, team routines in clinical settings

A related discussion is rapidly emerging in the fields of Human-Robot Interaction and Human-Autonomy Teaming, exploring how the introduction of AI agents influences intragroup processes in human teams (19). Conflicting findings regarding the correlation between AI adoption and teamwork performance in human-only vs human-AI teams highlight the need to gain deeper understanding of intragroup routines and AI teaming as subject to mutual change.

Teaming with agentic AI often means disrupting team routines. A socio-organizational view of routines shows they are not only fixed and bureaucratic. The implementation of AI changes according to pre-deployment organizational routines and team relations, and these routines and relations also change with the implementation of AI. Routines (like morning routines) embody both administrative/organizational and social/personal patterns of actions. We are only beginning to understand the formalized and negotiated aspects of *routine-making* in human-AI teaming, including the effects of intervening variables such as team processes (distributed/hierarchical communication and leadership, team diversity in terms of ranks and specialties, routine formalization vs negotiability) as well as the AI adaptability – e.g., automation and augmentation features that are used wholly, partially or not at all, by deployers along different phases in the workflow cycle (20–22).

As the new EU AIA takes effect, AI systems that mimic how human teams collaborate might improve explainability in high-risk situations of clinical medicine. This suggestion is an important move forward given that much of the research has so far focused on dyadic relationships between a human user and AI or treated the “team” as a unified unit (23). Our own on-going ethnographic study of the deployment of clinical AI in hospitals that follow FDA regulation problematizes this notion of mimicking how human teams collaborate by showing how AI-human teaming is negotiated vis-a-vis the spectrum of (in)formality of routines, from credentialing, licensing and reporting to putting together a medical performance that withstands trials of accountability. When routines are well-defined and formalized (“protocols”), teams are more likely to integrate the AI tool into their workflow, for example in allowing AI to autonomously detect and alert strokes from ICU scans; in contexts where routines are negotiated and interactions are voluntary, such as intragroup communication, AI’s agentic features, such as the documenting feature, might be restrained or avoided, for example not using the chat option to record consultations between clinicians.

## 5 The way forward

Medical progress in clinical AI requires parallel evolution of regulatory frameworks and clinical practice workflows. The precautionary nature of the regulation of AI in healthcare creates an experimental space where healthcare managers and physician deployers/users engage in configuring a usable framework for AI during an early adoption phase.

To become a sustainable approach in medical AI implementation, such “use first, trust later” deploying mindset we described cannot be left unmonitored. Major policy implications thus include the following:

(1) Regulation should ensure AI developers disclose their training data and model architecture for full transparency.

(2) Specific mechanisms could include mandating the use of shared data registries, across different demographic groups, for collaborative monitoring and evaluations of system performance by AI vendors, healthcare deployers, patient groups (especially those most likely to be harmed by these technologies) and independent auditing bodies. This would require a process of education and certification among these stakeholders.

(3) Hospital administrators and healthcare deployers should evaluate teaming routines with AI, and inform AI developers.

(4) Explainable AI should be developed within practical medico-legal framings of accountability. A clear disclosure, and even choice, regarding the trade-off between explainability and accuracy in specific healthcare AI systems should be provided to patients.

Striking a balance between regulation and beneficial use, accuracy and explainability, formal and negotiated AI-human teaming, requires empirical and interdisciplinary efforts. The challenge of post-deployment AI oversight in healthcare does not belong to one group such as either AI vendors or healthcare deployers, but involves a diverse range of stakeholders, including AI manufacturers, healthcare providers, patients - especially those from historically underrepresented groups - and regulatory bodies, fostering a system that is constantly learning and consistently adaptive.

## Data Availability

The original contributions presented in this study are included in this article/supplementary material, further inquiries can be directed to the corresponding author.
